# *Klebsiella quasipneumoniae* Provides a Window into Carbapenemase Gene Transfer, Plasmid Rearrangements, and Patient Interactions with the Hospital Environment

**DOI:** 10.1128/AAC.02513-18

**Published:** 2019-05-23

**Authors:** Amy J. Mathers, Derrick Crook, Alison Vaughan, Katie E. Barry, Kasi Vegesana, Nicole Stoesser, Hardik I. Parikh, Robert Sebra, Shireen Kotay, A. Sarah Walker, Anna E. Sheppard

**Affiliations:** a Division of Infectious Disease and International Health, Department of Medicine, University of Virginia Health System, Charlottesville, Virginia, USA; b Clinical Microbiology Laboratory, Department of Pathology, University of Virginia Health System, Charlottesville, Virginia, USA; c Modernizing Medical Microbiology Consortium, Nuffield Department of Clinical Medicine, University of Oxford, Oxford, United Kingdom; d NIHR Health Protection Research Unit in Healthcare Associated Infection and Antimicrobial Resistance at University of Oxford in partnership with Public Health England, Oxford, United Kingdom; e Health Information & Technology, University of Virginia Health System, Charlottesville, Virginia, USA; f School of Medicine Research Computing, University of Virginia, Charlottesville, Virginia, USA; g Icahn Institute, Department of Genetics and Genomic Sciences, Icahn School of Medicine, Mount Sinai, New York, New York, USA

**Keywords:** environmental reservoir, infection control, KPC, *Klebsiella*, *Klebsiella pneumoniae* carbapenemase, *Klebsiella quasipneumoniae*, sink drains, carbapenemase, multidrug resistance, premise plumbing

## Abstract

Several emerging pathogens have arisen as a result of selection pressures exerted by modern health care. Klebsiella quasipneumoniae was recently defined as a new species, yet its prevalence, niche, and propensity to acquire antimicrobial resistance genes are not fully described. We have been tracking inter- and intraspecies transmission of the Klebsiella pneumoniae carbapenemase (KPC) gene, *bla*_KPC_, between bacteria isolated from a single institution. We applied a combination of Illumina and PacBio whole-genome sequencing to identify and compare *K. quasipneumoniae* from patients and the hospital environment over 10- and 5-year periods, respectively. There were 32 *bla*_KPC_-positive *K. quasipneumoniae* isolates, all of which were identified as K. pneumoniae in the clinical microbiology laboratory, from 8 patients and 11 sink drains, with evidence for seven separate *bla*_KPC_ plasmid acquisitions. Analysis of a single subclade of *K. quasipneumoniae* subsp. *quasipneumoniae* (*n* = 23 isolates) from three patients and six rooms demonstrated seeding of a sink by a patient, subsequent persistence of the strain in the hospital environment, and then possible transmission to another patient. Longitudinal analysis of this strain demonstrated the acquisition of two unique *bla*_KPC_ plasmids and then subsequent within-strain genetic rearrangement through transposition and homologous recombination. Our analysis highlights the apparent molecular propensity of *K. quasipneumoniae* to persist in the environment as well as acquire carbapenemase plasmids from other species and enabled an assessment of the genetic rearrangements which may facilitate horizontal transmission of carbapenemases.

## Abstract (precis)

Several emerging pathogens have arisen as a result of selection pressures exerted by modern health care. Klebsiella quasipneumoniae was recently defined as a new species, yet its prevalence, niche, and propensity to acquire antimicrobial resistance genes are not fully described.

## INTRODUCTION

In the last 50 years, transformations in health care have created new niches for microorganisms such as Acinetobacter baumannii complex and Candida auris to arise from obscurity and emerge as important pathogens. Similarly, we have seen an increasing number of highly resistant Klebsiella pneumoniae strains which have been successfully transmitted worldwide (1). Klebsiella pneumoniae has proven to be an important contributor to the modern antibiotic resistance epidemic, with its ability to acquire and carry antimicrobial resistance plasmids, as well as being successful as a human pathogen. More recently, whole-genome sequencing has revealed that many isolates classified as K. pneumoniae actually encompass three related but distinct species: K. pneumoniae, Klebsiella variicola, and Klebsiella quasipneumoniae (1, 2). K. quasipneumoniae was originally thought to be largely confined to agriculture and the environment; however, it appears that it may also be prominent in human disease (3), and several recent reports have demonstrated that it harbors virulence factors and acquires clinically relevant genes of antimicrobial resistance (4, 5). Although there have been relatively few reports of K. quasipneumoniae to date, the true prevalence of this organism is likely underestimated as it is not generally distinguished from K. pneumoniae in routine testing of clinical laboratories (2).

Bacterial evolution via horizontal gene transfer is central to the ongoing crisis of antimicrobial resistance among clinically relevant bacteria. Hospital wastewater is being increasingly recognized as an ideal reservoir for resistance gene exchange and amplification, with ongoing antimicrobial selection pressure exerted through antimicrobials excreted in patient waste (6). Premise plumbing can be seeded by antimicrobial resistance genes in diverse bacterial strains and species and represents a difficult-to-treat reservoir for ongoing gene exchange, creating successful drug-resistant bacteria that can thrive in both the environmental and human niches (7).

Whole-genome sequencing studies have demonstrated that our understanding of the interplay between antimicrobial resistance plasmids and their host strains/species is limited (8). The host range of a plasmid is critical for acquisition and persistence in specific species, but it appears that some bacterial strains are better equipped than others to prevent the acquisition of or destroy foreign plasmid DNA (9). The durability of plasmid acquisition events and the creation of new highly resistant strains reflect complex dynamics which depend on the characteristics of the plasmid in question as well as host strain tolerance (10, 11). Seldom do we have the opportunity to witness strains acquiring plasmids *in vivo* or in the environment, and inferences about genetic rearrangements are often highly speculative. However, understanding the mechanisms and frequency of resistance gene transfer events occurring in real world contexts can provide important insights into the wider evolutionary landscape creating modern multidrug-resistant bacteria which cannot be effectively modeled in lab experiments (12).

Within our institution, we have seen ongoing transmission of diverse carbapenemase-producing organisms for the last decade, driven by genetic exchange of the Klebsiella pneumoniae carbapenemase (KPC) gene (*bla*_KPC_) in patients and the environment (13, 14). This has enabled us to understand specific pathways of genetic mobility involving numerous different mobile genetic elements and host bacterial species (13, 15). Herein, we examine *bla*_KPC_ acquisition and associated genetic rearrangements within *K. quasipneumoniae* as a real-life representation of an emerging pathogen associated with the hospital wastewater environment.

## RESULTS

**FIG 1:**
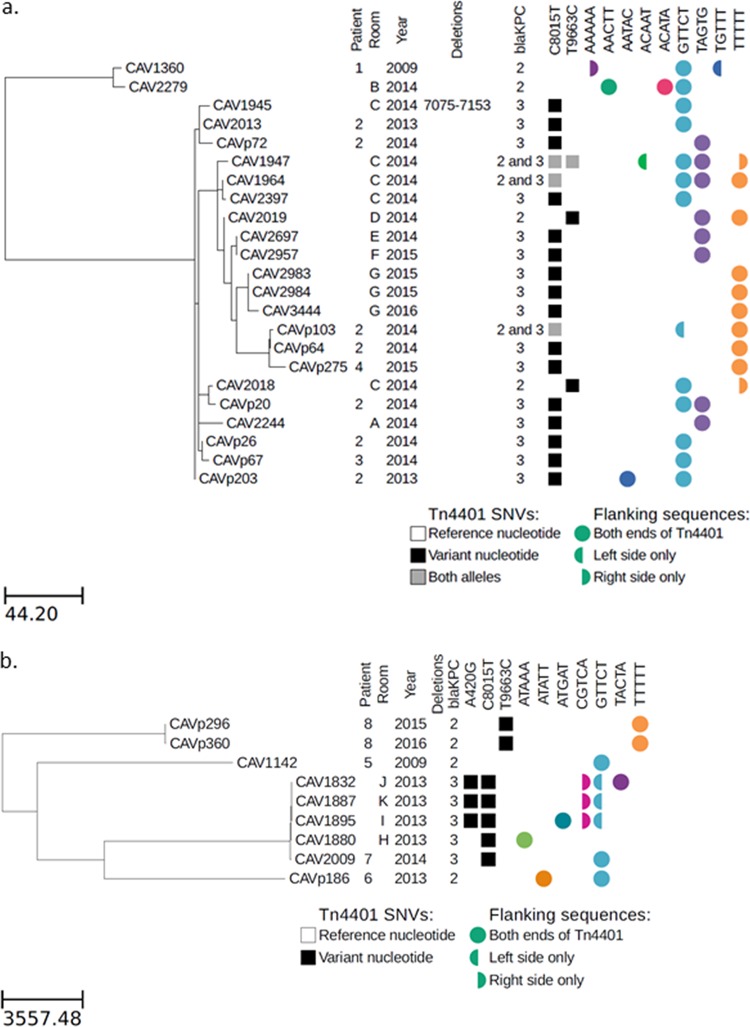
Maximum likelihood phylogeny for KpIIA (a) and KpIIB (b) isolates, with Tn*4401* variation and flanking genetic contexts. Branch lengths are shown as SNVs per genome.

From our collection of *bla*_KPC_-positive isolates from patients (2007 to 2017) and the hospital environment (2013 to 2017), there were a total of 32 *bla*_KPC_-positive K. quasipneumoniae isolates, all of which were identified as K. pneumoniae in the clinical microbiology laboratory (Table 1). Twenty-three of these were K. quasipneumoniae subspecies *quasipneumoniae* (KpIIA) (10 patient isolates from four patients and 13 environmental isolates from seven rooms), and nine were K. quasipneumoniae subspecies *similipneumoniae* (KpIIB) (five patient isolates from four patients and four environmental isolates from four rooms). The KpIIA and KpIIB isolates were separated by >100,000 single nucleotide variants (SNVs). We identified a single strain of KpIIA and four strains of KpIIB differing from each other by >20,000 SNVs (Fig. 1). Many isolates have multiple virulence factors (see Data Set S1 in the supplemental material), including several genes involved in capsule production (16) and several fimbrial elements. A type VI secretion system was present in all KpIIA but not all KpIIB isolates. From a resistance gene standpoint, in addition to *bla*_KPC_, all isolates harbored *fosA* and *bla*_OKP_ as well as a multidrug efflux transporter (*oqxA-oqxB*) (17).

**TABLE 1:** All Sequenced *bla*_KPC_-Klebsiella quasipneumoniae isolates from patients and the hospital environment

Label	Isolate	Subspecies of *K. quasipneumoniae*	Date (mo-yr)	Source	Infection/outcome
1	CAV1360	KpIIA	Nov-09	Sputum	Ventilator-associated pneumonia in complicated heart transplant recipient/expired
2	CAV2013	KpIIA	Nov-13	Perirectal surveillance	NA^*a*^
2	CAVp203	KpIIA	Dec-13	Bronchoscopy	Ventilator-associated pneumonia/successful treatment
2	CAVp26	KpIIA	Apr-14	Blood	Intraabdominal infection/expired
2	CAVp20	KpIIA	Mar-14	Perirectal surveillance	NA
2	CAVp64	KpIIA	Aug-14	Perirectal surveillance	NA
2	CAVp72	KpIIA	Sep-14	Perirectal surveillance	NA
2	CAVp103	KpIIA	Nov-14	Blood	Successful treatment
3	CAVp67	KpIIA	Aug-14	Perirectal surveillance	NA
4	CAVp275	KpIIA	Jul-15	Urine	Complicated urinary tract infection/successful treatment
5	CAV1142	KpIIB	Aug-09	Perirectal surveillance	NA
6	CAVp186	KpIIB	Dec-13	Perirectal surveillance	NA
7	CAV2009	KpIIB	Feb-14	Perirectal surveillance	NA
8	CAVp296	KpIIB	Oct-15	Perirectal surveillance	NA
8	CAVp360	KpIIB	Dec-16	Perirectal surveillance	NA
Room A (MICU^*b*^)	CAV2244	KpIIA	Jan-14	Shower	
Room B (CTA)	CAV2279	KpIIA	Jan-14	Shower	
Room C (STBICU^*c*^)	CAV1945	KpIIA	Feb-14	Drain swab (first sample after replacement)	
Room C (STBICU)	CAV1947	KpIIA	Feb-14	P-trap water (first sample after replacement)	
Room C (STBICU)	CAV1964	KpIIA	Mar-14	Drain swab	
Room C (STBICU)	CAV2018	KpIIA	Apr-14	P-trap water	
Room D (STBICU)	CAV2019	KpIIA	Apr-14	P-trap water	
Room C (STBICU)	CAV2397	KpIIA	May-14	Drain swab	
Room E (STBICU)	CAV2697	KpIIA	Jul-14	Drain swab	
Room F (MICU)	CAV2957	KpIIA	Sep-15	Drain swab	
Room G (STBICU)	CAV2983	KpIIA	Oct-15	P-trap water	
Room G (STBICU)	CAV2984	KpIIA	Oct-15	Drain swab	
Room G (STBICU)	CAV3444	KpIIA	Feb-16	P-trap water	
Room H (MICU)	CAV1880	KpIIB	Dec-13	Drain swab	
Room I (MICU)	CAV1895	KpIIB	Dec-13	Drain swab	
Room J (STBICU)	CAV1832	KpIIB	Dec-13	P-trap water	
Room K (STBICU)	CAV1887	KpIIB	Dec-13	P-trap water	

a NA, not applicable.

b MICU, medical intensive care unit.

c STBICU, surgical, trauma, and burn intensive care unit.

Within the KpIIA strain, there were two subclades separated by ∼150 SNVs (Fig. 1a). The first subclade contained two isolates separated by 10 SNVs (Fig. 1a). CAV1360 was from patient 1 in November 2009, and CAV2279 was identified in early 2014 (shortly after environmental sampling began) from room B that patient 1 had occupied in May 2009.

**FIG 2:**
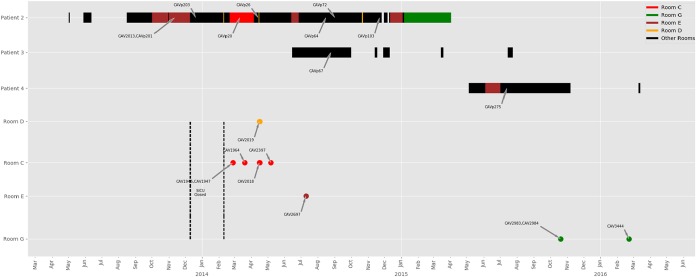
Patient movements and positive environmental samples with a single strain of *K. quasipneumoniae* (KpIIA) in the STBICU. Colored bars for patients match rooms where environmental isolates were identified. Black bars represent rooms with no KpIIA identified. The dotted lines indicate STBICU closure with removal and new installation of sink drains and exposed sink plumbing. Patient 1 is not depicted, as there was no admission to the STBICU and no overlap in time or space with other patients carrying KpIIA.

The second subclade of KpIIA contained isolates from three patients (patients 2 to 4) and six rooms (rooms A and C to G). The earliest of these was from patient 2 in November 2013. Patient 2 was in the hospital with a prolonged stay in the surgical, trauma, and burn intensive care unit (STBICU) following complications of a liver transplant (Fig. 2). Patient 2 was noted to be first colonized with *bla*_KPC_-positive KpIIA in November 2013. KpIIA was not found in the STBICU environment prior to closure for remediation of KPC contamination of the drains in December 2013. Following drain exchange and unit reopening, patient 2 was immediately moved back into the STBICU and subsequently occupied rooms C, D, E, and G in the STBICU, suggesting that the KpIIA isolates in these rooms originated from patient 2 (Fig. 2). Patient 3 was admitted to the STBICU at the same time as patient 2 and thus could have acquired KPC-KpIIA directly from patient 2 without environmental transmission. Patient 4 was later admitted to STBICU room E for 28 days and discharged before he was found to have KpIIA. He was never on a ward at the same time as any other patients known to carry KpIIA, suggesting acquisition from the hospital environment.

There were four patients (patients 5 to 8) carrying four distinct strains of *bla*_KPC_-KpIIB seen over a 5-year period (Fig. 1b, Table 1). For patient 7, the same KpIIB strain (∼80 SNV differences) was also seen in sinks from two rooms in the medical intensive care unit (MICU) (rooms H and I) and two rooms in the STBICU (rooms J and K) in December 2013 when environmental sampling first began; this preceded the detection in the patient in February 2014. Patient 7 was admitted to the MICU (location of rooms H and I), but did not stay any of the rooms where the isolates within the same KpIIB were identified. The other three patients with KpIIB each had unique *bla*_KPC_ strains, none of which were identified in another patient or the environment. Patient 6 also with a unique strain had a prolonged hospital stay and was also colonized/infected with another *bla*_KPC_-positive species (K. pneumoniae).

Three patients developed infections with KPC-KpIIA (Table 1). Patient 1 died of ventilator-associated pneumonia with KPC-KpIIA following a complicated heart transplant. Patient 2 had both ventilator-acquired pneumonia, which was successfully treated, and a subsequent untreatable intraabdominal infection with KPC-KpIIA bacteremia, which contributed to the patient’s death after a long hospital stay with a complicated liver transplant. Patient 4 had a successfully treated complicated KPC-KpIIA urinary tract infection. Patient 3 did not develop an infection with KpIIA. None of the patients with KpIIB developed *K. quasipneumoniae* infections; however, two of the patients did develop infections with other species carrying *bla*_KPC_ (K. pneumoniae for patient 6 and Serratia marcescens for patient 8) (Table 2).

**TABLE 2:** All additional *bla*_KPC_-positive isolates from patients with *K. quasipneumoniae*

Patient	Isolate	Species	Date (mo-yr)	Source	Infection	Genetic information (Tn*4401* isoform)	Flank sequence(s) (right/left)
2	CAVp202	S. marcescens	Dec-13	Urine	No	Tn*4401*b-8	TTTTT/TTTTT
2	CAVp11	S. marcescens	Feb-14	Intraabdominal abscess	Yes	Tn*4401*b-8	TTTTT/TTTTT
2	CAV1761	S. marcescens	Mar-14	Perirectal surveillance		Tn*4401*b-8	TTTTT/TTTTT
3	CAVp50	Klebsiella pneumoniae	Jul-14	Perirectal surveillance	NA^*a*^	Tn*4401*b-truncated (deletion 9299–10006)	—/TTGCA
3	CAVp57	Klebsiella pneumoniae	Jul-14	Perirectal surveillance	NA	Tn*4401*b-truncated	—/TTGCA
3	CAVp71	Klebsiella pneumoniae	Aug-14	Perirectal surveillance	NA	Tn*4401*b-truncated	—/TTGCA
3	CAVp104	Klebsiella pneumoniae	Dec-14	Perirectal surveillance	NA	Tn*4401*b-truncated	—/TTGCA
6	CAV1750	Klebsiella pneumoniae	Dec-12	Perirectal surveillance	NA	Tn*4401*b-1	GTTCT/GTTCT
6	CAVp127	Klebsiella pneumoniae	Feb-13	Perirectal surveillance	NA	Tn*4401*b-1	GTTCT/GTTCT
6	CAVp130	Klebsiella pneumoniae	Mar-13	Urine	Yes	Tn*4401*b-1	GTTCT/GTTCT
6	CAVp139	Klebsiella pneumoniae	Apr-13	Perirectal surveillance	NA	Tn*4401*b-1	GTTCT/GTTCT
6	CAVp151	Klebsiella pneumoniae	Jul-13	Perirectal surveillance	NA	Tn*4401*b-1	GTTCT/GTTCT
6	CAVp152	Klebsiella pneumoniae	Jul-13	Perirectal surveillance	NA	Tn*4401*b-1	GTTCT/GTTCT
6	CAVp177	Klebsiella pneumoniae	Sep-13	Perirectal surveillance	NA	Tn*4401*b-1	GTTCT/GTTCT
6	CAVp180	Klebsiella pneumoniae	Nov-13	Perirectal surveillance	NA	Tn*4401*b-1	GTTCT|TACCT/AGCAT|GTTCT
6	CAVp183	Klebsiella pneumoniae	Nov-13	Intraabdominal abscess	Yes	Tn*4401*b-1	GTTCT/GTTCT
6	CAVp184	Klebsiella pneumoniae	Nov-13	Perirectal surveillance	NA	Tn*4401*b-1	GTTCT/GTTCT
6	CAVp185	Klebsiella pneumoniae	Nov-13	Perirectal surveillance	NA	Tn*4401*b-1	ATATT|GTTCT/ATATT|GTTCT
6	CAVp3	Klebsiella pneumoniae	Jan-14	Biliary drain	Yes	Tn*4401*b-1	GTTCT/GTTCT
8	CAVp269	Serratia marcescens	Jun-15	Blood	Yes	Tn*4401*b-8	TTTTT/TTTTT
8	CAVp270	Serratia marcescens	Jun-15	Perirectal surveillance	NA	Tn*4401*b-8	TTTTT/TTTTT
8	CAVp361	Escherichia coli	Dec-16	Perirectal surveillance	NA	Tn*4401*b-8	TTTTT/TTTTT
8	CAVp374	Citrobacter freundii	Mar-17	Perirectal surveillance	NA	Tn*4401*b-8	TTTTT/TTTTT

a NA, not applicable.

### Genetic variation and rearrangements within KpIIA.

All KpIIA isolates were closely related at the core chromosome level, with a maximum divergence of <180 SNVs. If *bla*_KPC_ was acquired only once in this lineage, then any sequence variation within the 10-kb *bla*_KPC_ transposon Tn*4401* would be the result of mutational change, which is expected to be rare. Surprisingly, the Illumina sequence data revealed a great deal of sequence variation within Tn*4401* (Fig. 1a). Two sites (positions 8015 and 9663 in the Tn*4401*b reference) showed variation at the single nucleotide level, and one isolate had a deletion at positions 7075 to 7153. Interestingly, several isolates showed mixtures at one or both of the variable sites, indicating two or more different versions of Tn*4401* in the same isolate. This included mixtures at position 8015, which is located within the *bla*_KPC_ gene and differentiates *bla*_KPC-2_ and *bla*_KPC-3_, indicating that there were isolates with both *bla*_KPC_ alleles.

Similarly, if a single *bla*_KPC_ plasmid was acquired and stably maintained within KpIIA, then we would expect to see a single flanking sequence context for Tn*4401*. On the contrary, there was significant diversity in Tn*4401* flanking regions, with eight and seven different 5-bp sequences on the left and right sides of Tn*4401*, respectively, suggesting active transposition of Tn*4401* within KpIIA and/or multiple plasmid acquisitions.

To better understand the origin of the genetic diversity within and surrounding Tn*4401*, we performed long-read PacBio sequencing on three of the KpIIA isolates (CAV2013 from patient 2, CAV1947 from room C, and CAV2018 from room C), as well as a S. marcescens isolate from patient 2 (CAV1761). The room C isolates were chosen because this room only became positive after the admission of patient 2 following sink trap exchange in the STBICU; hence, they are expected to be descended from the patient 2 KpIIA.

**FIG 3:**
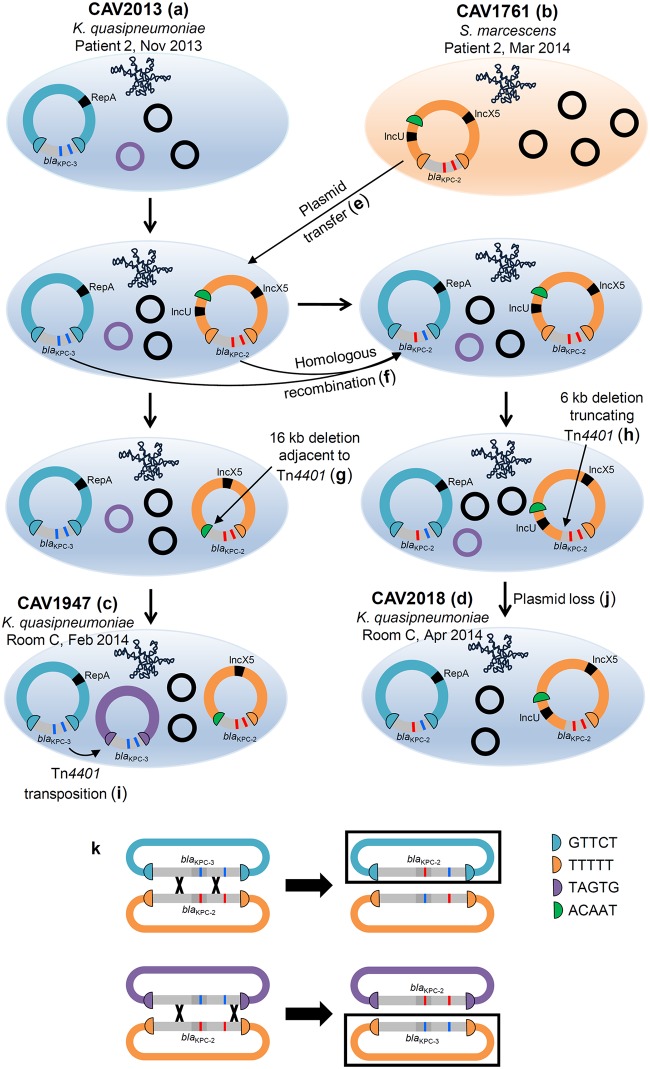
Plasmid structures determined from long-read sequencing of four isolates and inferred intermediate *bla*_KPC_ plasmid structures. (a to d) Sequenced isolates. (e to j) Inferred intermediate plasmid structures. Note that the ordering of deletion, homologous recombination, transposition, and plasmid loss events is arbitrarily represented, as the actual order of events is unknown. (k) Examples of crossover events leading to the generation of new combinations of SNVs within Tn*4401* (top) or the complete swapping of Tn*4401* variants between different plasmids (bottom). Black boxes indicate products of homologous recombination that were observed in long-read data (top) or Illumina data (bottom).

Both patient 2 isolates had a single *bla*_KPC_ plasmid each (Fig. 3a and b). The KpIIA isolate had a 447,095-bp “RepA_CP011611” *bla*_KPC-3_ plasmid, and the S. marcescens isolate had a 69,158-bp IncU/IncX5 *bla*_KPC-2_ plasmid (18). Both plasmids contained Tn*4401*b; however, there were two SNV differences within the Tn*4401*b sequence, one at position 8015 (differentiating *bla*_KPC-2_ and *bla*_KPC-3_) and one at position 9663.

**FIG 4:**
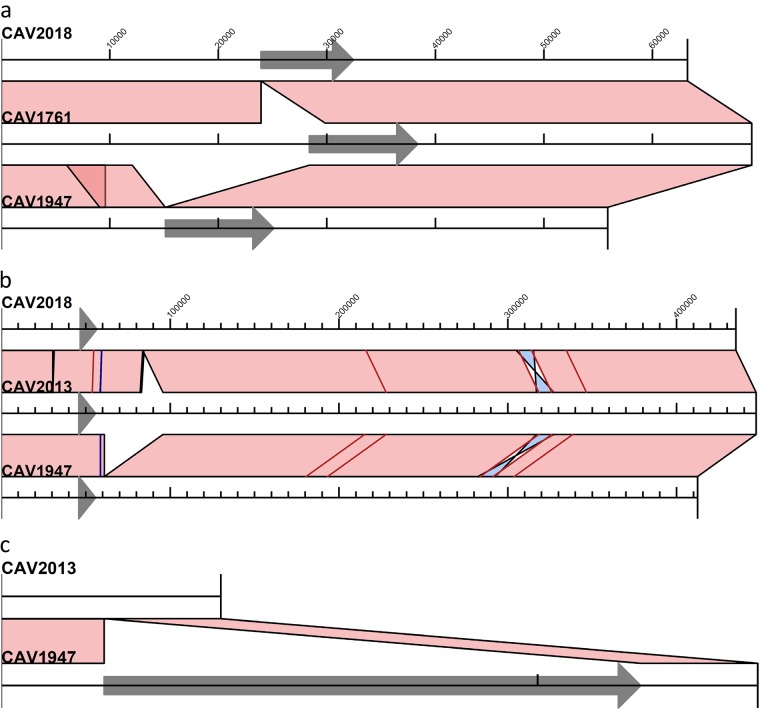
Alignments of IncU/IncX5 (a), RepA_CP011611 (b), and nontypeable (c) *bla*_KPC_ plasmid structures determined from long-read sequencing. Tn*4401* is indicated by a gray arrow. Light pink shading indicates regions of identity, light blue shading shows inverted regions, and SNVs are indicated by red lines and short indels by blue lines.

The KpIIA isolates from room C (CAV1947 and CAV2018) had three and two *bla*_KPC_ plasmids, respectively (Fig. 3c and d). Both isolates harbored the IncU/IncX5 *bla*_KPC_ plasmid from the patient 2 S. marcescens isolate, indicating likely *bla*_KPC_ plasmid transfer from S. marcescens to *K. quasipneumoniae* (Fig. 3e). In CAV1947, the plasmid sequence was identical to that from the patient isolate, CAV1761, with the exception of two large indels (Fig. 4a). One of these was a 16,315-bp deletion immediately adjacent to Tn*4401*, presumably as a result of intramolecular transposition in *cis*, that converted the left flanking sequence from TTTTT to ACAAT and removed the IncU replicon sequence (Fig. 3g). In CAV2018, the plasmid sequence was identical to that in CAV1761, except for a single 5,923-bp deletion that truncated part of the Tn*4401* sequence (Fig. 3h and 4a).

Both isolates also harbored the ancestral RepA_CP011611 *bla*_KPC_ plasmid from the patient 2 KpIIA isolate, with several SNVs and large indels (Fig. 4b). Interestingly, in CAV2018, one of the SNVs was located within Tn*4401*, such that the CAV2018 RepA_CP011611 plasmid contained *bla*_KPC-2_ rather than *bla*_KPC-3_. Given that there was plasmid transfer of the IncU/IncX5 *bla*_KPC-2_ plasmid from S. marcescens, we infer that the *bla*_KPC-2_-containing RepA_CP011611 plasmid most likely arose as a result of homologous recombination between these two different plasmids flanking the *bla*_KPC_ region (Fig. 3f and k). The Illumina data also revealed similar patterns of homologous recombination in other isolates (notably CAV2983, CAV2984, CAV3444, CAVp64 and CAVp275, which all have the TTTTT IncU/IncX5 plasmid flanking sequences, but with the C8015T *bla*_KPC-3_ mutation and without the T9663C mutation), suggesting frequent exchange of Tn*4401* variants between different *bla*_KPC_ plasmids within the same host bacterium (Fig. 1 and 3k).

CAV1947 also harbored a third *bla*_KPC_ plasmid, representing transposition of Tn*4401* into a 4,095-bp nontypeable plasmid that was present in the CAV2013 ancestor from patient 2 (Fig. 3i and 4c).

### *K. quasipneumoniae* has acquired *bla*_KPC_ on multiple occasions.

The average unique plasmid Inc types per isolate was more than four according to PlasmidFinder (Data Set S1). Within KpIIB, there were four divergent strains separated by >20,000 SNVs, suggesting four separate acquisitions of *bla*_KPC_ in this subspecies. Within KpIIA, there were two subclades separated by ∼180 SNVs. Given that Tn*4401* variation and flanking sequences were different between the two subclades (apart from the GTTCT flanking sequence which is known to be present in many different *bla*_KPC_ plasmids) (13) and that there was no epidemiological overlap, it is most likely that the subclades acquired *bla*_KPC_ independently. Additionally, as described above, the second subclade likely acquired *bla*_KPC_ on two occasions, with the second acquisition originating from S. marcescens. Therefore, overall, there were likely seven acquisitions of *bla*_KPC_ by *K. quasipneumoniae*: three in KpIIA and four in KpIIB.

Interestingly, there was evidence that one of the acquisitions in KpIIB also originated from S. marcescens, indicating the compatibility of these two species in exchanging plasmids. This was in the patient 8 KpIIB lineage. Patient 8 was first colonized with *bla*_KPC_-S. marcescens carrying Tn*4401*b with a T9663C mutation and TTTTT/TTTTT flanking sequences. Four months later, *bla*_KPC_-KpIIB was identified with the same Tn*4401* mutation and flanking sequences, suggesting plasmid transfer from S. marcescens to *K. quasipneumoniae* within this patient.

## DISCUSSION

We describe the behavior of nosocomial *bla*_KPC_-positive K. quasipneumoniae strains within a single hospital setting, observing their propensity to take up multiple carbapenemase plasmids from other species and disseminate between patients and sink drains. Our study also suggests that rapid genetic rearrangement occurs in the mobile genetic elements carrying *bla*_KPC_ in KpIIA.

There is increasing recognition that the hospital environment is an important reservoir in the transmission of carbapenemase-producing Enterobacteriaceae (CPE), but delineating transmission chains is often challenging (19, 20). Through our K. quasipneumoniae example, we provide compelling evidence for patient-to-drain and drain-to-patient transmission, as has been observed in other studies (7). We also provide evidence supporting the ability of K. quasipneumoniae to be maintained in the environment for a long period of time, with the first subclade of KpIIA detected in the environment on initial sampling, even though it had not been seen in a patient nor had that patient been in the room for more than 3 years. The costly closure of the STBICU and exchange of all the sink drain plumbing pipes had a limited effect on environmental contamination with CPE; instead, it appears to have provided an environment for immediate new seeding and establishment of previously unobserved carbapenem-resistant strains. There are potential other reservoirs to consider, but health care workers have not been identified as a source of CPE. We have a fairly robust screening program in place and have sequenced all patient isolates and included all K. quasipneumoniae in this series, making silent colonization less likely (21, 22). We were not sampling the toilets or hoppers during most of the study, and we have only sequenced a portion of environmental isolates which could provide an unidentified environmental source of K. quasipneumoniae (14). Understanding the dynamics and natural history of colonization of premise plumbing with CPE will be important in designing effective interventions to limit transmission (23).

Although there have only been a few reports of K. quasipneumoniae since its definition as a species in 2014, it appears that this organism is widespread (2, 5, 24, 25). As seen here, it is not readily distinguished from K. pneumoniae with current clinical microbiology techniques; thus, the true prevalence is unknown (2, 26). On the evolutionary time scale, modern medicine has provided a novel ecology with immunocompromised patients, widespread antimicrobial use, newly circulating antimicrobial resistance genes, and the design of the modern hospital providing new microbiologic niches for organisms to emerge (7, 27). We found several virulence factors in our collection, some of which have been identified in other K. pneumoniae or K. quasipneumoniae: capsule, fimbrial adhesion proteins, and a type VI secretion system (5, 16). As seen here, we provide evidence for *K. quasipneumoniae* to be sustained in both a human host and the environment, encountering several different species which may be relatively new in the evolutionary tree of *Klebsiella* sp. (1). As a consequence of these encounters, the transfer of mobile DNA occurs via traceable carbapenemase plasmids. We found evidence for seven independent acquisitions of *bla*_KPC_ by K. quasipneumoniae, suggesting that this species is amenable to take up plasmids from other species of Enterobacteriaceae. Given the difficulties in accurately identifying K. quasipneumoniae, this species may therefore be more significant in the context of *bla*_KPC_ dissemination than has previously been recognized.

Within K. quasipneumonia*e*, there was surprising variability in mobile elements carrying *bla*_KPC_, which was the result of several different processes observed among a limited number of highly related isolates (*n* = 23). We also found multiple acquired antimicrobial resistance genes, and every isolate had more than one plasmid incompatibility type (18). Specifically, there were multiple independent *bla*_KPC_ plasmid acquisitions: homologous recombination between different *bla*_KPC_ plasmids, transposition of Tn*4401* into new plasmids, intramolecular transposition in *cis* of Tn*4401*, a deletion within Tn*4401*, and a deletion truncating Tn*4401*. This high degree of genetic mobility has been similarly observed in other small studies (28, 29) and highlights the difficulty in developing an accurate understanding of the transmission epidemiology of important drug resistance genes which can be rapidly mobilized by multiple independent genetic modalities.

Within KpIIA, there were multiple acquisitions of *bla*_KPC_ within the same lineage, such that a *bla*_KPC_-positive KpIIA strain acquired a second unrelated *bla*_KPC_ plasmid from S. marcescens. Consequently, there were then two different *bla*_KPC_ plasmids, with different Tn*4401* sequences and different *bla*_KPC_ alleles, within the same host bacterium. This situation facilitated multiple rearrangements via homologous recombination between the different plasmids, resulting in the generation of new combinations of Tn*4401* SNVs and host plasmids. Multiple acquisition of resistance plasmids followed by rearrangements between those plasmids is likely to be important in the generation of adaptive allelic combinations which contribute to the amplification of cross-class antimicrobial resistance within strains. High-risk clones with a propensity to take up antimicrobial resistance plasmids may represent important targets for intervention (30).

This study has several limitations. Most notably, it is a small retrospective series, preventing a full understanding of the role of the environment. Also, the order of genetic rearrangements is also not completely known, given the limited number of long-read sequenced isolates and inability to capture all isolates from the environment over time. We offer, however, that this is higher resolution than seen in many studies, and the analysis contributes to the greater understanding of rapid rearrangement and mechanisms at play regarding the mobility of genetic elements harboring genes of antibiotic resistance in Enterobacteriaceae.

In summary, we demonstrate the relevance of K. quasipneumoniae as a species fit for nosocomial transmission in the modern era that is capable of acquiring and maintaining relevant resistance elements.

## MATERIALS AND METHODS

### Setting.

Isolates were collected at the University of Virginia, a 619-bed tertiary care hospital, from August 2007 to May 2017. A robust K. pneumoniae carbapenemase-producing organism (KPCO) prevention program existed throughout the study period, as previously described (31), and included perirectal screening beginning in April 2009 in the medical intensive care unit (MICU) and surgical intensive care unit (STBICU) and weekly screening of all patients in the MICU and STBICU as well as units where any known KPCO-colonized patient was present (32). Screening was performed as previously described (32). Clinical *Enterobacterales* and *Aeromonadaceae* isolates, as identified by matrix-assisted laser desorption ionization time of flight mass spectrometry (MALDI-TOF MS) or VITEK2 (bioMérieux, Durham, NC), with an elevated ertapenem or meropenem MIC by VITEK2 (bioMérieux, Durham, NC), immediately underwent CarbaR (Cepheid, Sunnyvale, CA) carbapenemase PCR testing. All species identification was performed using a combination of VITEK2 and Vitek-MS (bioMérieux, Durham, NC). Clinical data were gathered by retrospective electronic medical record review under University of Virginia Health Sciences institutional review board (IRB) number 13558 with waiver of consent.

In September 2013, sink trap sampling for KPCO began using previously described techniques (14) with a swab for drain collection and P-trap water. Following identification of KPCO in the hospital environment, the STBICU was closed to patient care in December 2013. Over the following 9 weeks, all sink drain pipes were removed and replaced with sink traps that eliminated overflows in the sink bowl. Patients were readmitted to the surgical intensive care unit in February 2014. Bleach, hydrogen peroxide, and ozone-impregnated water (2 ppm) were applied weekly from February to May 2014 in the STBICU (following drain exchange and sink bowl overflow closure and removal) and from March to May 2014 in the MICU (without drain exchange or sink bowl overflow removal).

### Whole-genome sequencing and bioinformatics analysis.

Illumina sequencing was performed as described previously (33). PacBio long-read sequencing and assembly were performed as previously described (13).

Broad-level species classification was performed using Kraken (34). To identify *K. quasipneumoniae* isolates, we queried all isolates initially classified as K. pneumoniae against reference sequences representing each of the four clades described by Holt et al. (1). We arbitrarily selected a single reference sequence for each clade; these were ERR025521 (KpI), ERR025986 (KpIIA), ERR025528 (KpIIB), and ERR025573 (KpIII). We used mash v1.1.1 (35) with parameters “-r -m 5” to compare Illumina data for each of our isolates to these reference sequences. Each isolate was then assigned to one of the four K. pneumoniae clades according to the reference with the lowest distance value. All isolates assigned to KpIIA or KpIIB were included in the analysis. In addition, we also included any other KPCO isolates from patients carrying *K. quasipneumoniae*.

To identify chromosomal single nucleotide variants (SNVs), Illumina reads for each *K. quasipneumoniae* isolate were mapped to the CAV2013 chromosome sequence (derived from long-read sequencing), with mapping and variant calling performed as described previously (36). A phylogeny was generated using IQ-TREE v1.3.13 (37) from an alignment of variable sites where at least 70% of samples had a high-quality reference/variant call (i.e., we excluded sites where >30% of samples had an “N” call). This was run with parameters “-blmin 0.000000001 -nt 4 -m GTR,” with -fconst used to specify the number of invariant sites.

To identify Tn*4401* variation and flanking sequences from Illumina data, we used TETyper with published parameters (38).

The Illumina paired-end short reads were *de novo* assembled using SPAdes assembler v 3.10.1 (35). Assembly statistics were evaluated using QUAST v4.0. (36) Plasmid Inc typing was performed using PlasmidFinder v2.0.1 against the Feb 2018 version of the Enterobacteriaceae database (16), with minimum identity of 80% and minimum coverage of 50%. Acquired antimicrobial resistance genes were screened from the assemblies using NCBI’s AMRFinder tool v1.0, which relies on a curated AMR protein database and a collection of hidden Markov models, with 90% minimum identity to translated amino acid residues and 50% minimum coverage of reference protein sequence. (37) Identification of bacterial virulence genes was performed using ABRicate v0.8.11 (https://github.com/tseemann/abricate), against the Virulence Factors Database (accessed on Feb 2019), with 80% minimum identity and 50% minimum coverage.

### Data availability.

Illumina paired-end sequence data can be accessed from NCBI BioProject identifier (ID) PRJNA411762. The accession numbers for completed closed genomes from hybrid assembly of PacBio and Illumina are GCA_003146655.1 (CAV2013), GCA_003146685.1 (CAV1947), GCA_003146635.1 (CAV2018), and GCA_003146705.1 (CAV1761). All other relevant data for the manuscript are within Data Set S1 in the supplemental material.

## Supplementary Material

Supplemental file 1
